# Pharmacological treatment for methamphetamine withdrawal: A systematic review and meta‐analysis of randomised controlled trials

**DOI:** 10.1111/dar.13511

**Published:** 2022-07-21

**Authors:** Liam S. Acheson, Benjamin H. Williams, Michael Farrell, Rebecca McKetin, Nadine Ezard, Krista J. Siefried

**Affiliations:** ^1^ The National Drug and Alcohol Research Centre UNSW Sydney Sydney Australia; ^2^ Alcohol and Drug Service St Vincent's Hospital Sydney Sydney Australia; ^3^ The National Centre for Clinical Research on Emerging Drugs c/o UNSW Sydney Sydney Australia; ^4^ Department of Psychiatry Royal Prince Alfred Hospital, Sydney Local Health District Sydney Australia; ^5^ New South Wales Drug and Alcohol Clinical Research and Improvement Network Sydney Australia

**Keywords:** drug therapy, methamphetamine, substance‐related disorder, systematic review, withdrawal

## Abstract

**Issues:**

Cessation of methamphetamine use may result in a characteristic withdrawal syndrome, no medication has been approved for this indication. This systematic review aims to assess the efficacy of pharmacotherapy for methamphetamine withdrawal, the first comprehensive meta‐analysis since 2008.

**Approach:**

MEDLINE (1966–2020), CINAHL (1982–2020), PsychINFO (1806–2020) and EMBASE (1947–2020) were systematically searched. Studies were included if they were randomised controlled trials (RCT) investigating pharmacological treatments for methamphetamine withdrawal, reviewing outcomes of treatment discontinuation, mental health outcomes, withdrawal symptoms (including craving) and patient safety. The relative risk (RR) and weighted mean difference (MD) were used to meta‐analyse dichotomous and continuous data respectively, with 95% confidence intervals. Risk of bias and Grading of Recommendations, Assessment, Development, and Evaluation (GRADE) assessments were conducted.

**Key Findings:**

Nine RCTs of six medications (*n* = 242 participants) met inclusion criteria, however, only six trials of four medications (*n* = 186) could be meta‐analysed. Mean sample size across studies was 27 participants, and 88% of participants were male. The quality of evidence in this review varies from low to very low on GRADE assessments. Amineptine may reduce discontinuation rates (RR 0.22, 95% confidence interval [CI] 0.07, 0.72, *p* = 0.01), and improve global state (MD −0.49, 95% CI −0.80, −0.17), compared with placebo, however, this medication is no longer approved. No other medications improved any domain when compared with placebo. Due to lack of reporting safety profiles could not be established.

**Conclusions:**

There is insufficient evidence to indicate any medication is effective for the treatment of methamphetamine withdrawal. The poor quality of the evidence indicates a need for better powered, high‐quality trials.

**Key points**

- We systematically reviewed nine randomised controlled trials of 242 participants, and meta‐analysed the results of six of those trials investigating four medications for the treatment of methamphetamine withdrawal.
- No medication displayed convincing results for the treatment of methamphetamine withdrawal, however, there is insufficient evidence to conclusively rule out any medication trialled to date due to poor quality evidence.
- Future research should focus on better powered, high‐quality trials to develop a comprehensive evidence base.

## 1 INTRODUCTION

Abrupt cessation of long‐term or chronic methamphetamine use can result in a clinically significant syndrome that involves dysphoric mood with at least two of the following symptoms: fatigue, vivid or unpleasant dreams, insomnia or hypersomnia, increased appetite, and psychomotor retardation or agitation [1]. The course of methamphetamine withdrawal is characterised by an early ‘crash’ phase (12–24 h) of exhaustion and fatigue, followed by a withdrawal phase (2–4 weeks) [2, 3, 4], with symptoms peaking within the first 7 days [2]. A protracted extinction phase (6–12 months), including cognitive deficits and affective symptoms has been described [5, 6, 7, 8, 9]. Withdrawal symptoms can be sufficiently severe to cause discomfort and relapse to use [10, 11, 12, 13]. Withdrawal management is therefore an imperative prerequisite to the effective treatment of methamphetamine dependence.

Methamphetamine use disorder is assosciated with significantly elevated mortality, increased incidence of HIV and hepatitis C infection, poor mental health (suicidality, psychosis, depression) and increased risk of cardiovascular events [14]. This has a substancial impact on clinical services and harms have grown over recent years [15, 16]. The United States has seen a significant increase in overdose deaths associated with stimulants (predominantly methamphetamine) over the last decade [17, 18], and in Australia the number of treatment episodes for methamphetamine use have increased since 2003 [19], leading to over 4000 presentations for methamphetamine withdrawal management annually [20].

Currently, there are no evidence‐based pharmacological or psychosocial treatments for methamphetamine withdrawal [4, 21]. The most recent Cochrane systematic review and meta‐analyses of pharmacological treatments was conducted in 2008, and identified four randomised controlled trials (RCT) investigating two pharmacological agents; amineptine and modafinil [4]. That review concluded there was no evidence to support either medication as a treatment for methamphetamine withdrawal. Narrative reviews conducted since then have reached similar conclusions and identify a serious gap in the literature to be addressed [22, 23, 24]. In the absence of clear evidence, treatment guidelines typically recommend supportive care and cautious symptomatic management with medications, such as diazepam and olanzapine with limited evidence of efficacy in this context [25, 26, 27].

Here, we conduct a systematic review and meta‐analyses to update the evidence for the efficacy of pharmacological treatments for methamphetamine withdrawal. Efficacy of the reviewed pharmacotherapies is compared to placebo for outcomes of treatment discontinuation, mental health outcomes, withdrawal symptoms (including craving) and patient safety.

## 2 METHODS

### 2.1 Protocol and registration

The protocol for this review was prospectively registered on the PROSPERO international prospective register of systematic reviews (CRD42021224850). We followed the Preferred Reporting Items for Systematic reviews and Meta‐Analyses (PRISMA) guidelines for reporting [28].

### 2.2 Included studies

RCT investigating a pharmacological intervention for methamphetamine withdrawal in humans were included in this review. Trial participants must have been diagnosed with methamphetamine or amphetamine use disorder or withdrawal, by standardised criteria (either the Diagnostic and Statistical Manual of Mental Disorders or International Classification of Diseases, any version). Participants who used substances other than methamphetamine/amphetamine were included, provided the primary drug of concern was methamphetamine or amphetamine and the inclusion criteria of the study did not require concurrent dependence or use of another substance. Any pharmacological intervention was included, with or without treatment as usual and with or without concurrent psychological treatment. Studies had to include a control arm that did not receive the intervention. Control groups with placebo, treatment as usual and/or psychosocial therapy were all included, provided both arms received the same adjuvant treatments.

### 2.3 Search methods

We searched four electronic databases (MEDLINE [1964 to December 2020], PsycINFO [1967–December 2020], CINAHL [1961–December 2020] and EMBASE [1974 to December 2020]). See Figure S1 for example search strategy. We also searched clinicaltrials.gov (to December 2020) for ongoing trials and hand‐searched the reference lists of all included papers for further studies. No language restrictions were placed on the search.

### 2.4 Study selection

Two reviewers (Liam S. Acheson and Benjamin H. Williams) independently assessed all titles and abstracts for relevance in Covidence® [29], and assessed full texts of relevant studies for inclusion. Discrepancies were resolved by discussion, and when no agreement could be reached a third reviewer (Nadine Ezard) made the final decision. The corresponding author was contacted if information necessary to assess a studies eligibility was not available in the original report.

### 2.5 Data extraction

Data were independently extracted by two authors (Liam S. Acheson and Benjamin H. Williams) into a structured data extraction template in Excel (Table S1). The template was piloted independently by two reviewers (Liam S. Acheson and Benjamin H. Williams) with one record prior to formal data extraction.

Data extracted included study characteristics, population descriptors, outcome data that aligned with our review outcomes, and details on study funding and location.

### 2.6 Outcomes

Outcomes were selected based on the commonly used measures and clinical indicators of treatment success in studies reporting on withdrawal treatments. The outcomes analysed were:

1. Discontinuation rate, measured by number of participants who did not complete the trial intervention.
2. Average score in global state as measured by a global psychiatric rating scale, for example, Clinical Global Impression of Change [30].
3. Average score in withdrawal symptoms as measured by withdrawal symptomology assessments, for example, Amphetamine Withdrawal Questionnaire [31].
4. Average score in craving as measured by craving rating scales, for example, Questionnaire for Evaluating Cocaine Craving and Related Responses [32], Visual Analog Scale [33].
5. Patient safety as measured by adverse events, serious adverse events, treatment‐related adverse events, study withdrawals.

### 2.7 Assessment of risk of bias and certainty of evidence

We assessed the methodological quality of included studies as per the Cochrane Risk of Bias tool Version 2 (RoB 2), [34, 35]. Outcomes studies were also reviewed using the Grading of Recommendations, Assessment, Development, and Evaluation (GRADE) approach to assess the certainty of evidence related to: risk of bias, inconsistency, indirectness and imprecision. Those with two or more reports of a single medication are reported in the main text and others reported in supplementary files [35, 36].

### 2.8 Data synthesis and analysis

Data from eligible studies for which sufficient data was available or provided were meta‐analysed, both in terms of any pharmacotherapy and a subgroup analysis of specific pharmacotherapy, to determine the level of effect of treatment options. If data were not included in the publication (i.e., means or SDs of reported results), attempts to contact the authors to obtain data were made.

Random effects meta‐analyses were conducted to account for within‐ and between‐study variance. For dichotomous data the relative risk (RR) and the 95% confidence intervals (CI) were reported and weighted by sample according to the Mantel–Haenszel method; for continuous data the standardised mean difference (SMD) and the 95% CI were reported and weighted according to the inverse variance of effect estimate. Pooled effects were presented in a forest plot and a *p*‐value of 0.05 was considered statistically significant. Sensitivity analysis was conducted for studies that used multiple measures for assessing withdrawal symptoms.

A narrative review of studies that met eligibility criteria, but were not included in the meta‐analyses was also conducted.

All meta‐analyses were conducted in Review Manager (RevMan) Version 5.4.1 for Windows [37].

## 3 RESULTS

### 3.1 Included studies

**FIGURE 1:**
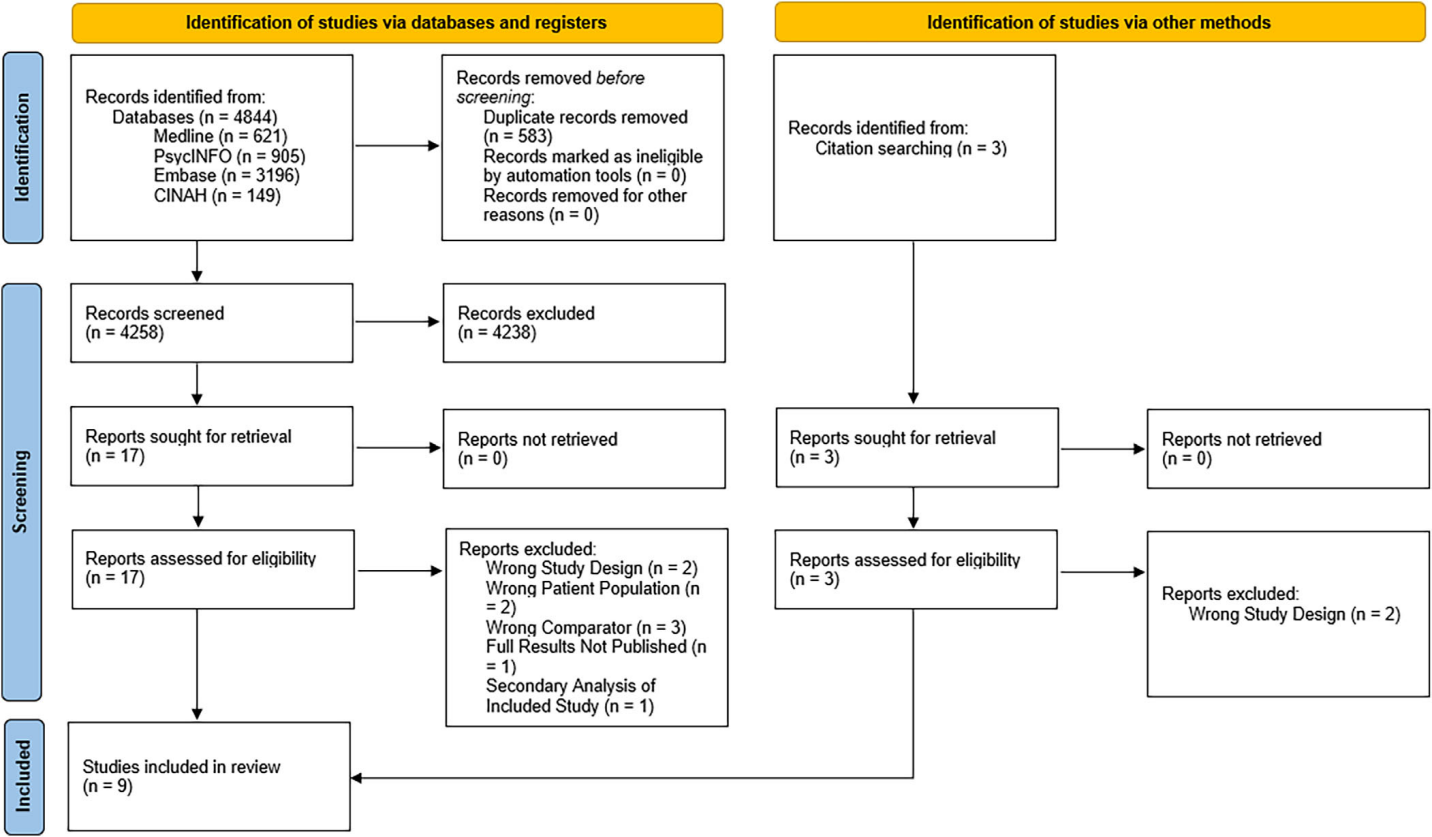
Preferred Reporting Items for Systematic reviews and Meta‐Analyses flow diagram *Source*: Adapted from Reference [28]. For more information, visit http://www.prisma‐statement.org/

Our search yielded 20 results, 11 of which did not meet inclusion criteria for this review [38, 39, 40, 41, 42, 43, 44, 45, 46, 47, 48]. Nine studies, including 242 participants met inclusion criteria (Figure 1). Table 1 presents the characteristics of the included studies. In total, 129 (48%) participants received a pharmacological treatment for methamphetamine/amphetamine withdrawal (22 mirtazapine, 27 modafinil, 5 ibudilast, 37 amineptine, 17 varenicline, 21 amantadine) and 138 (52%) received placebo. Studies were conducted in the United States (*n* = 3), Thailand (*n* = 3), Australia (*n* = 2) and Iran (*n* = 1). Mean sample size across studies was 27 participants, and approximately 88% were identified as male (one study did not report gender). Six of the nine included studies (67%) were meta‐analysed (186 participants, 89 receiving treatments [22 mirtazapine, 9 modafinil, 37 amineptine, 21 amantadine] and 97 receiving placebo) [49, 50, 51, 52, 53, 54]. Three studies were not meta‐analysed as although they met inclusion criteria, they did not report on the primary outcomes of this review [55, 56, 57].

**TABLE 1:** Included study characteristics

Author, year	Setting	Participants (*n*)	Participants who reached primary endpoint, *n* (%)	Gender, *n* (%) male	Age, mean (SD) years	Baseline MA use, mean (SD) days per month	Intervention, duration and comparison	Adjunctive intervention provided to both arms?
Cruikshank et al. 2008	Australia: Public, inner‐city drug and alcohol out‐patient settings	31	16 (53%)	20 (63%)	31 (1)	22.4 (1.6)	Mirtazapine, 15 mg nocte OD for 2 nights then 30 mg nocte OD for 12 nights. Placebo controlled	Narrative therapy counselling, 5 sessions per week, of 45 min (maximum 10 sessions over treatment period)
Lee et al. 2013	Australia: Short term metropolitan residential withdrawal units	19	11 (58%)	12 (65%)	34.3 (SD not reported)	18.3 (8.9)	Modafinil, 200 mg OD for 5 days then 100 mg for 2 days. Placebo controlled	Not reported
Birath et al. 2017	USA: Inpatient, residential research unit	11	^a^	Not reported	42.8 (SD not reported)	Not reported	Ibudilast, 10 mg BID or 25 mg BID for 5 days. Placebo controlled	Not reported
Jittiwutikan et al. 1997	Thailand: Inpatient, residential	30	22 (73%)	29 (97%)	18.5 (3.4)	Not reported	Amineptine, 150 mg BID for 14 days, titrated up over the first 5 days. Placebo controlled	Low dose lorazepam, supportive treatment, for example, nutrition
Kalechstein et al. 2014	USA: Residential research facility for treatment phases	26	^a^	18 (71%)	35.7 (2.1)	13.1 (1.5)	Varenicline, 0, 1 or 2 mg BID for 7 days, then crossover to other arm. Placebo controlled	Not reported
Kongsakon et al. 2005	Thailand: Specialised probation centre	20	16 (80%)	20 (100%)	24.3 (SD not reported)	Not reported	Mirtazapine, 15–60 mg OD titrated per clinical response for 14 days. Trial did not exceed 30 mg. Placebo controlled	None provided
Mahoney et al. 2012	USA: Inpatient, research centre	18	^a^	16 (89%)	34.8 (7.7)	18.3 (8.9)	Modafinil, one dose of 200 mg OD delivered on day 6 or 7 of the intervention, then counter condition the next day. Placebo controlled	None provided
Modaressi et al. 2018	Iran: Outpatient, with weekly visits to treatment facility	42	35 (83%)	42 (100%)	30.9 (4.7)	Not reported (participants >3 weeks abstinent)	Amantadine, 100 mg OD for 28 days. Placebo controlled	Symptomatic therapy
Srisurapanont et al. 1999	Thailand: Inpatient drug treatment centre	44	35 (80%)	41 (93%)	19.6 (5.5)	Not reported	Amineptine, 150 mg BID for 5 days, delivered in three 100 mg capsules given at breakfast and lunch. Placebo controlled	Low dose lorazepam, supportive treatment, for example, nutrition

Abbreviations: BID, twice daily; MA, methamphetamine; OD, once daily.

^a^ Human laboratory studies, participant retention not reported.

### 3.2 Comparisons

Of the nine eligible studies, all (100%) were placebo controlled, and six studies reported enough data to be meta‐analysed or provided the data when requested. Two studies examined amineptine [51, 54], two mirtazapine [49, 52], one amantadine [53] and one modafinil [50]. The remaining three studies which could not be meta‐analysed examined ibudilast [55], varenicline [56] or modafinil [57].

### 3.3 Risk of bias

**FIGURE 2:**
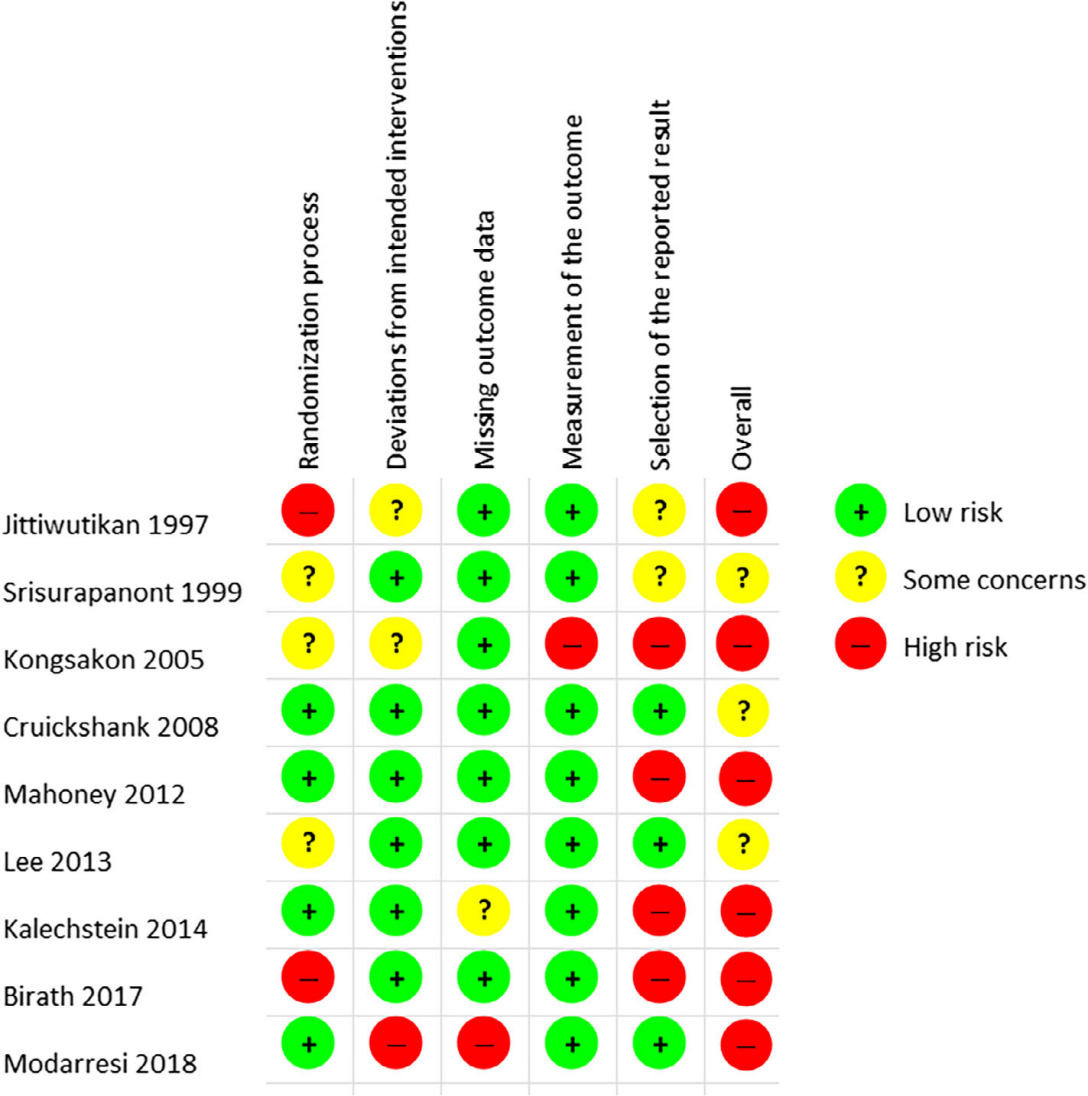
Methodological quality graph

Of the nine included studies, one study was judged to be of low risk of bias, two indicated some concerns and six indicated a high risk of bias (Figure 2). The most common area of bias identified was selective reporting of results, which affected six of the nine studies [51, 52, 54, 55, 56, 57], four of which were of high risk of bias [52, 55, 56, 57]. This was followed by issues in the randomisation process due to unclear randomisation processes or significant differences at baseline indicating issues with randomisation (five affected studies [50, 51, 52, 54, 55]) and deviations from intended intervention (three affected studies [51, 52, 53]). Two studies were judged to have risks surrounding missing outcome data [53, 56], and one for issues with measurement of the intervention as blinding of outcome assessors was unclear [52].

### 3.4 Effects of intervention

A summary of meta‐analysis results and GRADE assessment for all outcomes are presented in Table 2. Forrest plots of all analyses are available in Figure S2.

**TABLE 2:** Summary of meta‐analysis and GRADE assessment results

Outcome	Intervention	*N* studies	*N* participants	*I* ^2^	Differential statistic (95% CI)	GRADE rating
Discontinuation					Relative risk	
	Amineptine	2	74	58	0.22 (0.07, 0.72)*	⨁◯◯◯ Very low
	Mirtazapine	2	51	0	0.96 (0.48, 1.92)	⨁◯◯◯ Very low
	Modafinil	1	19	^a^	0.67 (0.22, 2.03)	^a^
	Amantadine	1	42	^a^	1.33 (0.34, 5.24)	^a^
	Total	6	186	18	0.70 (0.40, 1.23)	⨁◯◯◯ Very low
Global state					Mean difference	
	Amineptine	2	72	0	−0.49 (−0.80, −0.17)*	⨁⨁◯◯ Low
	Mirtazapine	1	31	^a^	0.30 (−0.22, 0.25)	^a^
	Total	3	103	69	−0.24 (−0.73, 0.25)	⨁◯◯◯ Very low
Withdrawal symptoms					Standardised mean difference	
	Amineptine	1	43	^a^	−0.26 (−0.86, 0.34)	^a^
	Mirtazapine	1	31	^a^	0.17 (−0.54, 0.89)	^a^
	Modafinil	1	19	^a^	0.86 (−0.09, 1.82)	^a^
	Total	3	93	49	0.17 (−0.43, 0.77)	⨁⨁◯◯ Low
Craving					Standardised mean difference	
	Amineptine	1	29	^a^	−0.19 (−0.92, 0.54)	^a^
	Modafinil	1	19	^a^	0.95 (−0.01, 1.92)	^a^
	Total	2	48	71	0.34 (−0.77, 1.45)	⨁◯◯◯ Very low
Safety					Relative risk	
	Amantadine	1	35	^a^	2.47 (0.76, 8.03)	^a^
	Total	1	35	^a^	2.47 (0.76, 8.03)	⨁◯◯◯ Very low

Abbreviations: CI, confidence interval; GRADE, Grading of Recommendations, Assessment, Development, and Evaluation.

^a^ Not calculated as only one study of the investigational product available.

* *p* < 0.05.

### 3.5 Discontinuation rates

In the six studies of 188 people included in meta‐analysis, the discontinuation rate was defined as the number of participants who did not remain enrolled in the trial at the primary end point. Overall, no difference in discontinuation rates was observed between treatment and placebo groups (RR 0.70, 95% CI 0.40, 1.23, *p* = 0.21).

In a subgroup analysis, amineptine was the only pharmacotherapy to show significant effect (RR 0.22, 95% CI 0.07, 0.72, *p* = 0.01). Heterogeneity was low and was not statistically significant across studies (*I* ^2^ = 18%, *p* = 0.30).

### 3.6 Global state

Three studies measured mental health by assessing global state: one study [49] used the Brief Symptom Inventory Global Severity Index [58], while the remaining two [51, 54] reported the Clinical Global Impression scale [30]. For both scales a higher score indicates greater symptom severity.

There was no difference in measures of global state between treatment and placebo (SMD −0.34, 95% CI −1.06, 0.25, *p* = 0.39), and the results were significantly and substantially heterogeneous (*I* ^2^ = 69%, *p* = 0.04).

In the subgroup analysis, participants receiving amineptine were significantly more likely to improve in Global State when compared with placebo (SMD −0.70, 95% CI −1.18, −0.22, *p* = 0.004). No difference was apparent for mirtazapine compared with placebo from a single study (SMD 0.42, 95% CI −0.30, 1.15, *p* = 0.36). All other trials were not included as they did not report on Global State.

### 3.7 Withdrawal symptoms

Four studies reported withdrawal outcomes, of which three (with 93 participants) were included in the meta‐analysis [49, 50, 54].

Studies assessed withdrawal symptoms using the Amphetamine Withdrawal Questionnaire (AWQ) [31], a 10‐item instrument based on the Diagnostic and Statistical Manual of Mental Disorders‐IV withdrawal criteria [54], and the Amphetamine Cessation Symptoms Assessment (ACSA) [49], a 16‐item questionnaire with items derived from the AWQ and the Cocaine Selective Severity Assessment [59]. One trial [50] reported withdrawal using three different measures, the AWQ, the ACSA as well as the Amphetamine Selective Severity Assessment, a modification of the Cocaine Selective Severity Assessment where the word ‘cocaine’ was replaced with the word ‘amphetamine’ [59, 60]. The AWQ data was reported for the purposes of this review, as the AWQ is the most common measure used for withdrawal severity in similar studies not included in the scope of this review [61].

There was no difference between treatment and placebo for withdrawal symptoms (SEM 0.17, 95% CI −0.43, 0.77, *p* = 0.58).

Sensitivity analysis was conducted where AWQ results were replaced with ACSA and Amphetamine Selective Severity Assessment values, and results remained insignificant overall and for modafinil alone (data not shown).

One trial which reported withdrawal outcomes was excluded from the meta‐analysis [52] as the data were reported as median, and the mean and SD required for comparison were not available or provided by request to authors. That study did, however, report significant improvement in withdrawal symptoms of participants receiving mirtazapine compared with placebo as measured by the AWQ (*p* = 0.03).

### 3.8 Cravings

Two studies (*n* = 48) reported cravings for methamphetamine in this review [50, 51]. A further trial included a craving measure, but this was conducted prior to pharmacological intervention with no follow‐up after the intervention commenced, so it was not included in this analysis [57]. Craving was assessed with the Questionnaire for Evaluating Cocaine Craving and Related Responses (QECCRR) [51], and a 100 mm Visual Analogue Scale (VAS) to evaluate craving for methamphetamine [50]. A VAS is a visual tool used to rate severity of symptoms [33]. In Lee et al. participants were asked to rate their ‘desire to use’ by putting a mark on a 100 mm long line indicating intensity of desire [50]. The QECCRR is a tool designed to measure four aspects of cocaine withdrawal through four 20 cm VASs: depressed mood, no energy, sick feeling and craving [32]. Only the craving score of the QECCRR was used in this analysis. As higher scores on the QECCRR indicate lower craving, and higher scores on the VAS indicate greater craving, the means of the QECCRR were multiplied by −1 to ensure consistency in direction of both scales.

There was no difference in treatment or placebo arms for craving scores (SMD 0.34, 95% CI −0.77, 1.45, *p* = 0.55) and data were substantially, but not significantly heterogenous (*I* ^2^ = 71%, *p* = 0.06). No individual medication alone showed effect.

### 3.9 Safety

Only one study included the number and type of adverse events (AE) experienced by participants receiving either amantadine or placebo, and found no significant differences between either arm (RR 2.47, 95% CI 0.76, 8.03, *p* = 0.13) [53]. Three studies described AEs in text. Lee et al. described no AEs in either the modafinil or placebo groups, but reported lethargy and disturbed sleep in both arms [50]. Kongsakon reported no serious adverse events during the trial of mirtazapine, but side effects were reported, including mild headache, nausea and vomiting [52]. Kalechstein stated that varenicline was not associated with neuropsychiatric or dose‐dependent adverse events, but gave no further information [56]. The four remaining studies did not include any information about safety or AEs [49, 54, 55, 57].

### 3.10 GRADE quality of evidence

The quality of evidence in this review varies from low to very low on GRADE assessments. Outcomes with multiple studies reporting on the same medications are presented in Table 2. For amineptine's effect on discontinuation rates we downgraded the evidence to very low, due to serious concerns with risk of bias and very serious concerns regarding imprecision. Mirtazapine's effect on discontinuation rates was similarly downgraded to very low due to very serious concerns regarding risk of bias and imprecision. For amineptine's effect on global state, we downgraded the evidence to low, due to serious concerns with risk of bias and imprecision. A full GRADE summary of findings table is available in Table S2.

### 3.11 Findings from studies not included in the meta‐analysis

Amantadine was found to reduce fatigue relative to placebo (*p* < 0.001, effect size not reported) [53]. A cross‐over study found modafinil significantly reduced sleep latency compared to baseline (*p* < 0.001, *η* ^2^ = 0.9), however, there was no difference between treatment and placebo after crossover (*p* > 0.05, effect size not reported) [57]. Two studies failed to produce improvements in sleep with mirtazapine (*p* > 0.05, effect sizes not reported), with one report finding increased awakenings throughout the night when compared to placebo (*p* < 0.05, effect size not reported) [49, 52].

Compared to placebo, varenicline was associated with significantly faster visual reaction time (*p* = 0.025, *η* ^2^ = 0.103), but did not demonstrate improvements on other neuropsychological tests, including working memory and sustained attention (*p* > 0.082, *η* ^2^ < 0.063) [56]. Ibudilast produced improvements in 2 of 12 domains on the Continuous Performance Test—II (variability [*p* < 0.01, *r* = 0.83] and perseverance [*p* = 0.01, *r* = 0.75]) which assesses sustained attention [55].

## 4 DISCUSSION

This review identified nine RCT assessing six medications for the treatment of amphetamine or methamphetamine withdrawal which met inclusion criteria. The results of this review indicate that there is insufficient evidence to support any pharmacotherapy as being effective for the treatment of methamphetamine withdrawal. However, the low to very low quality of available evidence means that neither is there sufficient evidence to confirm that any of the studied pharmacotherapies are not effective. Further high‐quality trials may result in different findings.

Withdrawal is commonly the first phase in reducing or ceasing regular methamphetamine use, and given that 96 people per 100,000 people globally are dependent on amphetamines, there is a clear need for high quality clinical trials in this space [62], the lack of which has been noted previously [22]. The last Cochrane review of this topic [4], which employed a similar search strategy to ours, was conducted in 2008. In the 13 years following that report, our search found only five additional RCT, and only two of those were able to be meta‐analysed. This is in stark contrast to other withdrawal syndromes where, for example, a recent Cochrane review of a single pharmacotherapy (buprenorphine) for opioid withdrawal yielded 27 RCT of 3048 participants [63].

### 4.1 Efficacy

No medication was found to be significantly different from placebo in terms of measures of discontinuation rates, global state, craving for amphetamine/methamphetamine or intensity of withdrawal symptoms. The lack of amelioration of any withdrawal symptoms in the studies reviewed here is of particular importance: withdrawal is often the first step in a treatment journey, and successful treatment of withdrawal symptoms can predict longer‐term treatment outcomes [12, 21]. The exception to these results was amineptine, which improved measures of global state and discontinuation when compared with placebo. However, this is of limited clinical utility, as amineptine was never approved for use in a variety of jurisdictions, including the United States, and was voluntarily withdrawn from the market by the manufacturer in 1999 due to reports of hepatotoxicity and amineptine abuse [64, 65]. Of any of the reviewed pharmacotherapies, amineptine's pharmacological and subjective effects may most closely mimic methamphetamine, in that it does have some immediate dopamine releasing effect, which may explain why positive effects were seen for this medication [66]. This suggests that like other withdrawal management strategies (e.g., tobacco, opioids) the strategy of pursuing a drug that has a similar (but longer acting) pharmacological profile to methamphetamine may be a more promising path for research going forward.

### 4.2 Safety

The safety profile of any of the reviewed medications is not well established in this population or context, as no safety data were reported in four of the trials, and in another four only a narrative description of adverse events was provided—making assessment of relative safety impossible. Only one trial systematically reported the number and type of adverse events experienced by each arm, and due to this no strong inference can be made about safety or side effects of the medications trialled in the included studies.

### 4.3 Sleep and cognition

The use of sleep as an outcome measure in clinical trials involving treatment for stimulant use is increasing. Reliable methods to objectively measure sleep outcomes should in future be harmonised across studies to allow for better synthesis of results. Further, the reliability of common sleep questionnaires has not been established in the context of people who consume drugs, and particularly in the case of stimulants, may have problematic wording. For example, the Insomnia Severity Index asks ‘How satisfied/dissatisfied are you with your current sleep pattern?’. A person who takes stimulants to intentionally stay awake might be very satisfied with not sleeping for days at a time, and this scale could therefore incorrectly assign healthy sleep patterns [67]. Studies in this review used a combination of objective measures and questionnaires assessing different aspects of sleep and due to these disparate outcomes and measures future pooled analysis of these data would be impossible.

Neither study assessing neurocognitive outcomes demonstrated robust evidence of efficacy. While varenicline suggested a non‐significant trend towards improved accuracy in the placebo group, and ibudilast found significant effect on two subscales of the 12‐part CPT‐II, both studies were judged to be of high risk of bias and so claims of efficacy based on these results are dubious. In addition, data are lacking on the neurocognitive impacts of methamphetamine withdrawal including the affected domains and severity, thus the clinical relevance of improvements in neurocognition by these measures in the acute methamphetamine withdrawal phase is yet to be established.

### 4.4 Limitations

This review has several limitations. By choosing to focus on only the highest level of evidence, RCT, this review did not include different study designs. However, due to the low quality of evidence in the trials reviewed here it is unlikely that the inclusion of uncontrolled studies or case reports would have changed the conclusions of the review. There is a need for updated, validated scales for assessing treatment outcomes in these populations, for example, the Clinical Global Impression was validated in 1977 [30].

Any implications for policy and practise from this review are limited by the quality and quantity of available data. Six of nine included papers were judged to be of high risk of bias in this review, limiting the reliability of results generated. The most likely reason for a high risk of bias assessment was apparent selective reporting of the outcome data and issues with randomisation. However, this review has highlighted the opportunity for future research as no large, well‐conducted clinical trial has yet been conducted, and there are potential avenues of investigation available. Future trials should be sufficiently powered and of high quality in both design and reporting, as further studies with methodological or reporting issues will not improve the level of evidence for pharmacological intervention for methamphetamine withdrawal in any meaningful way.

## 5 CONCLUSION

The results of this review did not find any of the reviewed medications to be efficacious in the treatment of amphetamine/methamphetamine withdrawal. Neither did it, however, find sufficient evidence to reject outright any of the previously trialled medications due to small sample sizes and methodological issues. There is a need and opportunity going forwards to conduct high impact research in pharmacological interventions for methamphetamine withdrawal. Future studies should provide consistent or harmonised outcome measures, accurate and detailed safety reporting and clear randomisation procedures. Study Registration: Prospero ID CRD42021224850.

## AUTHOR CONTRIBUTIONS

Each author certifies that their contribution to this work meets the standards of the International Committee of Medical Journal Editors.

## CONFLICT OF INTEREST

Michael Farrell as Director of NDARC has received unrestricted funding for research purposes from Indivior and Sequiiris. Liam S. Acheson is funded by a NDARC PhD Scholarship and NCCRED. No other authors have any conflicts to declare.

## Supporting information


**Figure S1** Example search

**Figure S2** Forrest plots of all analyses.

**Table S1** Structured data extraction template

**Table S2** Summary of findings table and GRADE rating for each outcome overall and for specific interventions when more than one study was included in the analysis

## References

[1] Diagnostic and statistical manual of mental disorders: DSM‐5. Fifth ed. Arlington, VA: American Psychiatric Publishing; 2013.

[2] McGregor C , Srisurapanont M , Jittiwutikarn J , Laobhripatr S , Wongtan T , White JM . The nature, time course and severity of methamphetamine withdrawal. Addiction. 2005;100:1320–9. 16128721 10.1111/j.1360-0443.2005.01160.x

[3] Newton TF , Kalechstein AD , Duran S , Vansluis N , Ling W . Methamphetamine abstinence syndrome: preliminary findings. Am J Addict. 2004;13:248–55. 15370944 10.1080/10550490490459915

[4] Shoptaw SJ , Kao U , Heinzerling K , Ling W . Treatment for amphetamine withdrawal. Cochrane Database Syst Rev. 2009;2:CD003021. 10.1002/14651858.CD003021.pub2 PMC7138250 19370579

[5] Wang G‐J , Volkow ND , Chang L , Miller E , Sedler M , Hitzemann R , et al. Partial recovery of brain metabolism in methamphetamine abusers after protracted abstinence. Am J Psychiatry. 2004;161:242–8. 14754772 10.1176/appi.ajp.161.2.242

[6] Iudicello JE , Woods SP , Vigil O , Cobb Scott J , Cherner M , Heaton RK , et al. Longer term improvement in neurocognitive functioning and affective distress among methamphetamine users who achieve stable abstinence. J Clin Exp Neuropsychol. 2010;32:704–18. 20198527 10.1080/13803390903512637 PMC2911490

[7] Salo R , Nordahl TE , Galloway GP , Moore CD , Waters C , Leamon MH . Drug abstinence and cognitive control in methamphetamine‐dependent individuals. J Subst Abus Treat. 2009;37:292–7. 10.1016/j.jsat.2009.03.004 PMC2739270 19339145

[8] Johanson C‐E , Frey KA , Lundahl LH , Keenan P , Lockhart N , Roll J , et al. Cognitive function and nigrostriatal markers in abstinent methamphetamine abusers. Psychopharmacology (Berl). 2006;185:327–38. 16518646 10.1007/s00213-006-0330-6

[9] Proebstl L , Krause D , Kamp F , Hager L , Manz K , Schacht‐Jablonowsky M , et al. Methamphetamine withdrawal and the restoration of cognitive functions – a study over a course of 6 months abstinence. Psychiatry Res. 2019;281:112599. 31629302 10.1016/j.psychres.2019.112599

[10] Meredith CW , Jaffe C , Ang‐Lee K , Saxon AJ . Implications of chronic methamphetamine use: a literature review. Harv Rev Psychiatry. 2005;13:141–54. 16020027 10.1080/10673220591003605

[11] Scott JC , Woods SP , Matt GE , Meyer RA , Heaton RK , Atkinson JH , et al. Neurocognitive effects of methamphetamine: a critical review and meta‐analysis. Neuropsychol Rev. 2007;17:275–97. 17694436 10.1007/s11065-007-9031-0

[12] Brecht M‐L , von Mayrhauser C , Anglin MD . Predictors of relapse after treatment for methamphetamine use. J Psychoactive Drugs. 2000;32:211–20. 10908010 10.1080/02791072.2000.10400231

[13] Hartz DT , Frederick‐Osborne SL , Galloway GP . Craving predicts use during treatment for methamphetamine dependence: a prospective, repeated‐measures, within‐subject analysis. Drug Alcohol Depend. 2001;63:269–76. 11418231 10.1016/s0376-8716(00)00217-9

[14] Farrell M , Martin NK , Stockings E , Bórquez A , Cepeda JA , Degenhardt L , et al. Responding to global stimulant use: challenges and opportunities. Lancet. 2019;394:1652–67. 31668409 10.1016/S0140-6736(19)32230-5 PMC6924572

[15] McKetin R , Degenhardt L , Shanahan M , Baker AL , Lee NK , Lubman DI . Health service utilisation attributable to methamphetamine use in Australia: patterns, predictors and national impact. Drug Alcohol Rev. 2018;37:196–204. 28294443 10.1111/dar.12518

[16] Jones CM , Houry D , Han B , Baldwin G , Vivolo‐Kantor A , Compton WM . Methamphetamine use in the United States: epidemiological update and implications for prevention, treatment, and harm reduction. Ann N Y Acad Sci. 2022;1508:3–22. 34561865 10.1111/nyas.14688 PMC9097961

[17] United Nations. World Drug Report. 2021. Contract No.: E.21.XI.8.

[18] Mattson CL , Tanz LJ , Quinn K , Kariisa M , Patel P , Davis NL . Trends and geographic patterns in drug and synthetic opioid overdose deaths—United States, 2013–2019. MMWR Morb Mortal Wkly Rep. 2021;70:202–7. 33571180 10.15585/mmwr.mm7006a4 PMC7877587

[19] McKetin R , Chrzanowska A , Man N , Peacock A , Sutherland R , Degenhardt L . Trends in treatment episodes for methamphetamine smoking and injecting in Australia, 2003–2019. Drug Alcohol Rev. 2021;40:1281–6. 33641198 10.1111/dar.13258

[20] Australian Institute of Health and Welfare . Alcohol and other drug treatment services. Canberra: AIHW; 2021.

[21] Lee NK , Rawson RA . A systematic review of cognitive and behavioural therapies for methamphetamine dependence. Drug Alcohol Rev. 2008;27:309–17. 18368613 10.1080/09595230801919494 PMC4445690

[22] Pennay AE , Lee NK . Putting the call out for more research: the poor evidence base for treating methamphetamine withdrawal. Drug Alcohol Rev. 2011;30:216–22. 21355922 10.1111/j.1465-3362.2010.00240.x

[23] Wodarz N , Krampe‐Scheidler A , Christ M , Fleischmann H , Looser W , Schoett K , et al. Evidence‐based guidelines for the pharmacological management of acute methamphetamine‐related disorders and toxicity. Pharmacopsychiatry. 2017;50:87–95. 28297728 10.1055/s-0042-123752

[24] Karila L , Weinstein A , Aubin HJ , Benyamina A , Reynaud M , Batki SL . Pharmacological approaches to methamphetamine dependence: a focused review. Br J Clin Pharmacol. 2010;69:578–92. 20565449 10.1111/j.1365-2125.2010.03639.x PMC2883750

[25] Härtel‐Petri R , Krampe‐Scheidler A , Braunwarth W‐D , Havemann‐Reinecke U , Jeschke P , Looser W , et al. Evidence‐based guidelines for the pharmacologic management of methamphetamine dependence, relapse prevention, chronic methamphetamine‐related, and comorbid psychiatric disorders in post‐acute settings. Pharmacopsychiatry. 2017;50:96–104. 28445899 10.1055/s-0043-105500

[26] Lintzeris N , Sunjic S , Demirkol A , Branezac M , Ezard N , Siefried K , et al. Management of withdrawal from alcohol and other drugs: an Evidence Check rapid review brokered by the Sax Institute (www.saxinstitute.org.au) for the NSW Ministry of Health. 2019.

[27] Grigg J , Manning V , Aurunogiri S , Volpe I , Frei M , Phan V , et al. Methamphetamine treatment guidelines: practice guidelines for health professionals. 2nd ed. Richmond, Victoria: Turning Point; 2018.

[28] Page MJ , McKenzie JE , Bossuyt PM , Boutron I , Hoffmann TC , Mulrow CD , et al. The PRISMA 2020 statement: an updated guideline for reporting systematic reviews. BMJ. 2021;372:n71. 33782057 10.1136/bmj.n71 PMC8005924

[29] Chappell D , Egger S . Australian violence: contemporary perspectives II. Canberra, Australia: Australian Institute of Criminology; 1995.

[30] Guy W . ECDEU assessment manual for psychopharmacology. Rockville, MD: US Department of Health, Education, and Welfare, Public Health Service; 1976.

[31] Srisurapanont M , Jarusuraisin N , Jittiwutikan J . Amphetamine withdrawal: I. Reliability, validity and factor structure of a measure. Aust N Z J Psychiatry. 1999;33:89–93. 10197890 10.1046/j.1440-1614.1999.00517.x

[32] Voris J , Elder I , Sebastian P . A simple test of cocaine craving and related responses. J Clin Psychol. 1991;47:320–3. 2030141 10.1002/1097-4679(199103)47:2<320::aid-jclp2270470221>3.0.co;2-f

[33] Lee JW , Brown ES , Perantie DC , Bobadilla L . A comparison of single‐item visual analog scales with a multiitem likert‐type scale for assessment of cocaine craving in persons with bipolar disorder. Addict Disord Their Treat. 2002;1:140–2.

[34] Sterne JA , Savović J , Page MJ , Elbers RG , Blencowe NS , Boutron I , et al. RoB 2: a revised tool for assessing risk of bias in randomised trials. BMJ. 2019;366:l4898. 31462531 10.1136/bmj.l4898

[35] The GRADE Working Group . GRADE handbook for grading quality of evidence and strength of recommendations. 2013. Available from: guidelinedevelopment.org/handbook

[36] Guyatt GH , Oxman AD , Vist GE , Kunz R , Falck‐Ytter Y , Alonso‐Coello P , et al. GRADE: an emerging consensus on rating quality of evidence and strength of recommendations. BMJ. 2008;336:924–6. 18436948 10.1136/bmj.39489.470347.AD PMC2335261

[37] Review Manager (RevMan) Version 541: The Cochrane Collaboration; 2020.

[38] Chan‐Ob T , Kuntawongse N , Boonyanaruthee V . Bupropion for amphetamine withdrawal syndrome. J Med Assoc Thail. 2001;84:1763–5. 11999825

[39] Cox D , Bowers R , McBride A . Reboxetine may be helpful in the treatment of amphetamine withdrawal. Br J Clin Pharmacol. 2004;58:100–1. 15207001 10.1111/j.1365-2125.2004.02094.x PMC1884544

[40] Johnson BA , Roache JD , Ait‐Daoud N , Wells LT , Wallace CL , Dawes MA , et al. Effects of topiramate on methamphetamine‐induced changes in attentional and perceptual‐motor skills of cognition in recently abstinent methamphetamine‐dependent individuals. Prog Neuro‐Psychopharmacol Biol Psychiatry. 2007;31:123–30. 10.1016/j.pnpbp.2006.08.002 PMC1810424 16978753

[41] McGregor C , Srisurapanont M , Mitchell A , Wickes W , White JM . Symptoms and sleep patterns during inpatient treatment of methamphetamine withdrawal: a comparison of mirtazapine and modafinil with treatment as usual. J Subst Abus Treat. 2008;35:334–42. 10.1016/j.jsat.2007.12.003 18329221

[42] Gillin JC , Pulvirenti L , Withers N , Golshan S , Koob G . The effects of lisuride on mood and sleep during acute withdrawal in stimulant abusers: a preliminary report. Biol Psychiatry. 1994;35:843–9. 8054406 10.1016/0006-3223(94)90019-1

[43] Kalechstein A , Mahoney J , Verrico CD , Iqbal T , De La Garza R . Varenicline improves information processing speed and verbal memory in methamphetamine‐dependent participants. Drug Alcohol Depend. 2015;100:e152.

[44] Solhi H , Jamilian HR , Kazemifar AM , Javaheri J , Barzaki AR . Methylphenidate vs. resperidone in treatment of methamphetamine dependence: a clinical trial. Saudi Pharm J. 2014;22:191–4. 25061402 10.1016/j.jsps.2013.04.003 PMC4099559

[45] Ahmadi J , Sahraian A , Biuseh M . A randomized clinical trial on the effects of bupropion and buprenorphine on the reduction of methamphetamine craving. Trials. 2019;20:468. 31362784 10.1186/s13063-019-3554-6 PMC6668115

[46] Ahmadi J , Razeghian JL . Comparing the effect of buprenorphine and methadone in the reduction of methamphetamine craving: a randomized clinical trial. Trials. 2017;18:259. 28587620 10.1186/s13063-017-2007-3 PMC5461765

[47] Mancino MJ , Thostenson JD , Guise JB , McGaugh J , Oliveto A . Impact of lisdexamfetamine on retention of methamphetamine‐dependent patients in a residential facility. Drug Alcohol Depend. 2017;171:e128.

[48] Hester R , Lee N , Pennay A , Nielsen S , Ferris J . The effects of modafinil treatment on neuropsychological and attentional bias performance during 7‐day inpatient withdrawal from methamphetamine dependence. Exp Clin Psychopharmacol. 2010;18:489–97. 21186923 10.1037/a0021791

[49] Cruickshank CC , Montebello ME , Dyer KR , Quigley A , Blaszczyk J , Tomkins S , et al. A placebo‐controlled trial of mirtazapine for the management of methamphetamine withdrawal. Drug Alcohol Rev. 2008;27:326–33. 18368615 10.1080/09595230801935672

[50] Lee N , Pennay A , Hester R , McKetin R , Nielsen S , Ferris J . A pilot randomised controlled trial of modafinil during acute methamphetamine withdrawal: feasibility, tolerability and clinical outcomes. Drug Alcohol Rev. 2013;32:88–95. 22630616 10.1111/j.1465-3362.2012.00473.x

[51] Jittiwutikan J , Srisurapanont M , Jarusuraisin N . Amineptine in the treatment of amphetamine withdrawal: a placebo‐controlled, randomised, double‐blind study. J Med Assoc Thail. 1997;80:587–92. 9347672

[52] Kongsakon R , Papadopoulos KI , Saguansiritham R . Mirtazapine in amphetamine detoxification: a placebo‐controlled pilot study. Int Clin Psychopharmacol. 2005;20:253–6. 16096515 10.1097/01.yic.0000166815.83017.d8

[53] Modarresi A , Eslami K , Kouti L , Hassanvand R , Javadi M , Sayyah M . Amantadine reduces persistent fatigue during post‐acute withdrawal phase in methamphetamine abstained individuals: a randomized placebo‐controlled trial. J Subst Use. 2018;23:584–90.

[54] Srisurapanont M , Jarusuraisin N , Jittiwutikan J . Amphetamine withdrawal: II. A placebo‐controlled, randomised, double‐blind study of amineptine treatment. Aust N Z J Psychiatry. 1999;33:94–8. 10197891 10.1046/j.1440-1614.1999.00518.x

[55] Birath JB , Briones M , Amaya S , Shoptaw S , Swanson A‐N , Tsuang J , et al. Ibudilast may improve attention during early abstinence from methamphetamine. Drug Alcohol Depend. 2017;178:386–90. 28704767 10.1016/j.drugalcdep.2017.05.016

[56] Kalechstein AD , Mahoney JJ 3rd , Verrico CD , De La Garza R 2nd . Short‐term, low‐dose varenicline administration enhances information processing speed in methamphetamine‐dependent users. Neuropharmacology. 2014;85:493–8. 24930359 10.1016/j.neuropharm.2014.05.045

[57] Mahoney JJ , Jackson BJ , Kalechstein AD , De La Garza R 2nd , Chang LC , Newton TF . Acute modafinil exposure reduces daytime sleepiness in abstinent methamphetamine‐dependent volunteers. Int J Neuropsychopharmacol. 2012;15:1241–9. 22214752 10.1017/S1461145711001805 PMC3411896

[58] Derogatis L . Brief symptom inventory administration, scoring, and procedures manual. Minneapolis: The Psychological Corporation, National Computer Systems. Inc; 1993.

[59] McGregor C , Srisurapanont M , Mitchell A , Longo MC , Cahill S , White JM . Psychometric evaluation of the amphetamine cessation symptom assessment. J Subst Abus Treat. 2008;34:443–9. 10.1016/j.jsat.2007.05.007 17629443

[60] Kampman KM , Volpicelli JR , Mcginnis DE , Alterman AI , Weinrieb RM , D'Angelo L , et al. Reliability and validity of the cocaine selective severity assessment. Addict Behav. 1998;23:449–61. 9698974 10.1016/s0306-4603(98)00011-2

[61] Siefried KJ , Acheson LS , Lintzeris N , Ezard N . Pharmacological treatment of methamphetamine/amphetamine dependence: a systematic review. CNS Drugs. 2020;34:337–65. 32185696 10.1007/s40263-020-00711-x PMC7125061

[62] Vos T , Abajobir AA , Abate KH , Abbafati C , Abbas KM , Abd‐Allah F , et al. Global, regional, and national incidence, prevalence, and years lived with disability for 328 diseases and injuries for 195 countries, 1990–2016: a systematic analysis for the Global Burden of Disease Study 2016. Lancet. 2017;390:1211–59. 28919117 10.1016/S0140-6736(17)32154-2 PMC5605509

[63] Gowing L , Ali R , White JM , Mbewe D . Buprenorphine for managing opioid withdrawal. Cochrane Database Syst Rev. 2017;2:CD002025. 28220474 10.1002/14651858.CD002025.pub5 PMC6464315

[64] Lazaros G , Stavrinos C , Papatheodoridis G , Delladetsima J , Toliopoulos A , Tassopoulos N . Amineptine induced liver injury. Report of two cases and brief review of the literature. Hepato‐Gastroenterology. 1996;43:1015–9. 8884331

[65] Perera I , Lim L . Amineptine and midazolam dependence. Singap Med J. 1998;39:129–31. 9632974

[66] Garattini S . Pharmacology of amineptine, an antidepressant agent acting on the dopaminergic system: a review. Int Clin Psychopharmacol. 1997;12:S15–20. 10.1097/00004850-199707003-00003 9347388

[67] Bastien CH , Vallières A , Morin CM . Validation of the insomnia severity index as an outcome measure for insomnia research. Sleep Med. 2001;2:297–307. 11438246 10.1016/s1389-9457(00)00065-4

