# Supplementary figures and images for: Pharmacological treatment for methamphetamine withdrawal: A systematic review and meta‐analysis of randomised controlled trials
Source: Drug Alcohol Rev. 2022 Jul 21;42(1):7–19. doi: 10.1111/dar.13511 (PMC10083934; doi:10.1111/dar.13511)

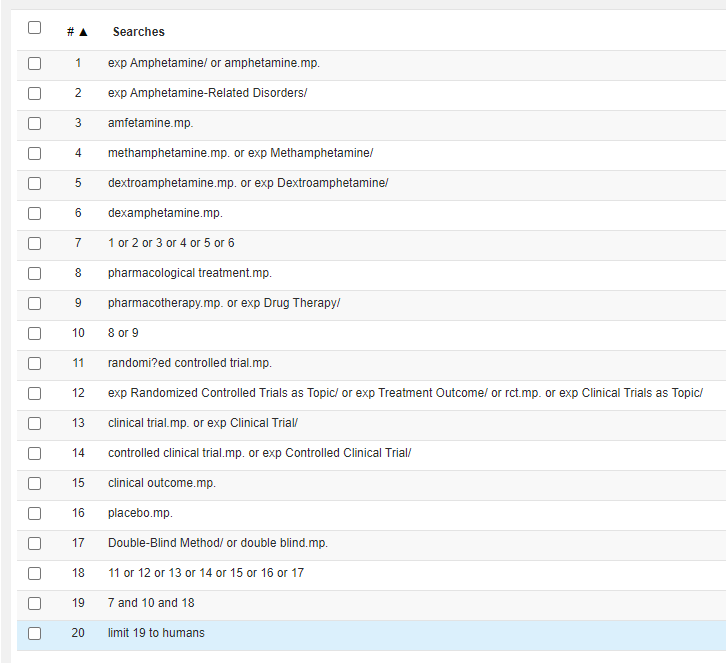

Supplement: Supplementary file 1 — Figure S1 Example search [file DAR-42-7-s001.png]
