# Supplementary material for: Pharmacological treatment for methamphetamine withdrawal: A systematic review and meta‐analysis of randomised controlled trials
Source: Drug Alcohol Rev. 2022 Jul 21;42(1):7–19. doi: 10.1111/dar.13511 (PMC10083934; doi:10.1111/dar.13511)

**Figure S2: Forrest plots of all analyses**

**S2.1 Forrest plot – Discontinuation rates**


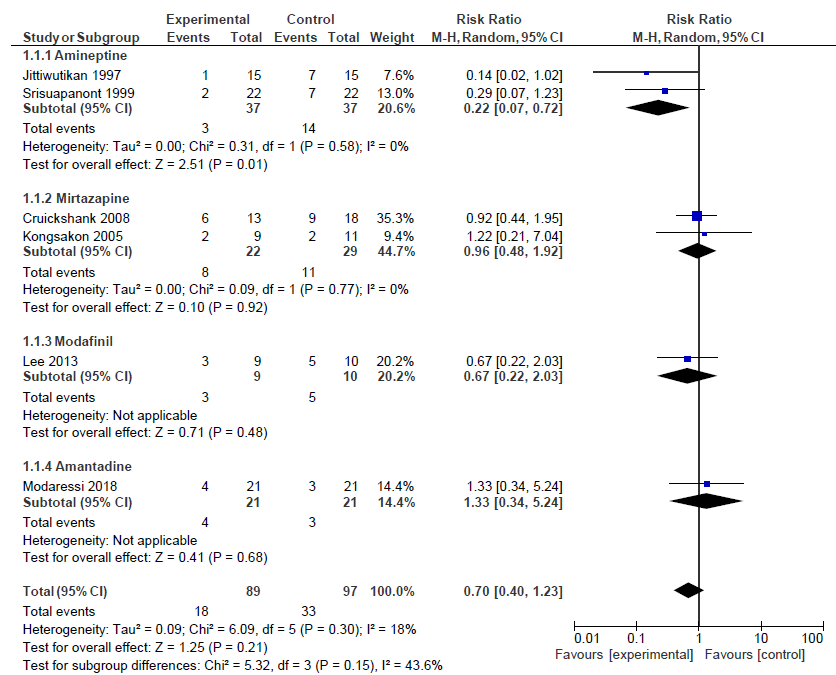


**S2.2 Forrest plot – Global state**


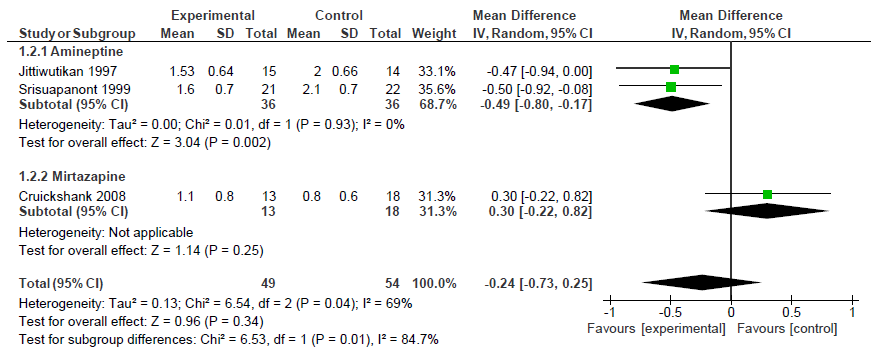


**3.3 Forrest plot – Withdrawal Symptoms**


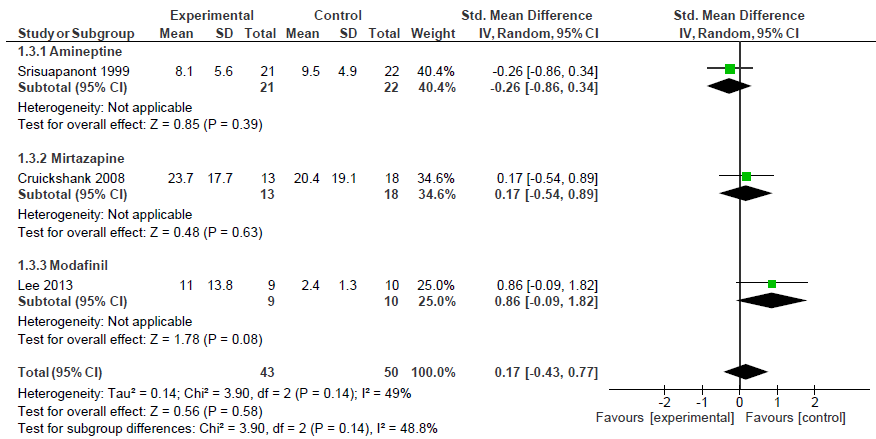


**3.4 Forrest Plot - Craving**


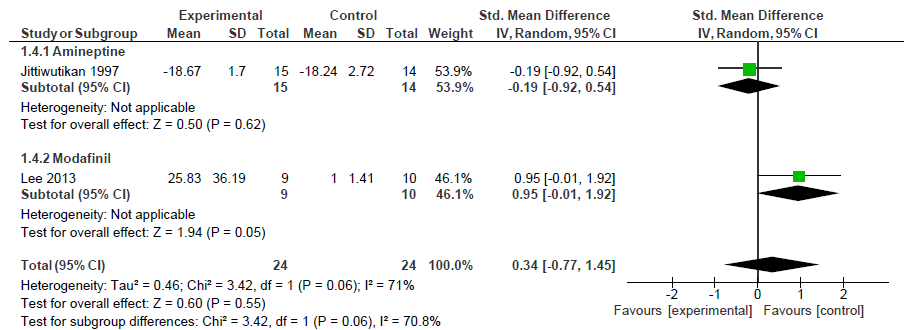


**3.5 Forrest Plot - Safety**


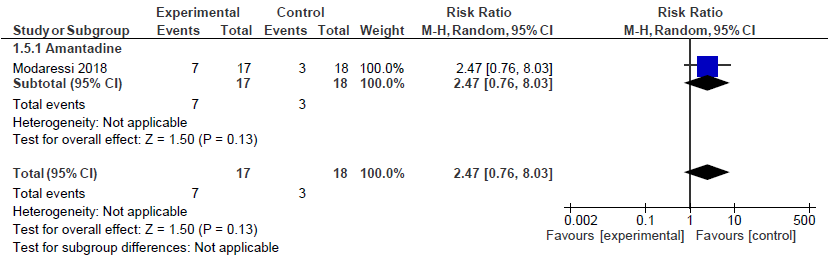

Supplement: Supplementary file 2 — Figure S2 Forrest plots of all analyses. [file DAR-42-7-s004.docx]
