# Supplementary material for: Pharmacological treatment for methamphetamine withdrawal: A systematic review and meta‐analysis of randomised controlled trials
Source: Drug Alcohol Rev. 2022 Jul 21;42(1):7–19. doi: 10.1111/dar.13511 (PMC10083934; doi:10.1111/dar.13511)
Supplement: Supplementary file 4 — Table S2 Summary of findings table and GRADE rating for each outcome overall and for specific interventions when more than one study was included in the analysis [file DAR-42-7-s002.docx]

**Table S2. Summary of findings table and GRADE rating for each outcome overall and for specific interventions when more than one study was included in the analysis**

| **Certainty assessment** | | | | | | | **№ of patients** | | **Effect** | | **Certainty** | **Importance** |
| --- | --- | --- | --- | --- | --- | --- | --- | --- | --- | --- | --- | --- |
| **№ of studies** | **Study design** | **Risk of bias** | **Inconsistency** | **Indirectness** | **Imprecision** | **Other considerations** | **Pharmacological intervention** | **Placebo** | **Relative (95% CI)** | **Absolute (95% CI)** |  |  |
| **Discontinuation rate** | | | | | | | | | | | | |
| 6 | Randomised trials | Very serious^a^ | Not serious | Not serious | Very serious^b^ | None | 18/89 (20.2%) | 33/97 (34.0%) | **RR 0.62** (0.38 to 1.01) | **129 fewer per 1000** (from 211 fewer to 3 more) | ⨁◯◯◯ Very low |  |
| *Discontinuation rate - Amineptine* | | | | | | | | | | | | |
| 2 | Randomised trials | Serious^c^ | Not serious | Not serious | Very serious^d^ | None | 3/37 (8.1%) | 14/37 (37.8%) | **RR 0.21** (0.07 to 0.69) | **299 fewer per 1000** (from 352 fewer to 117 fewer) | ⨁◯◯◯ Very low |  |
| *Discontinuation rate - Mirtazapine* | | | | | | | | | | | | |
| 2 | Randomised trials | Very serious^e^ | Not serious | Not serious | Very serious^b^ | None | 8/22 (36.4%) | 11/29 (37.9%) | **RR 0.98** (0.49 to 1.97) | **8 fewer per 1000** (from 193 fewer to 368 more) | ⨁◯◯◯ Very low |  |
| **Global state** | | | | | | | | | | | | |
| 3 | Randomised trials | Serious^c^ | Serious^f^ | Not serious | Very serious^b^ | None | 49 | 54 | - | MD **0.27 lower** (0.54 lower to 0.01 lower) | ⨁◯◯◯ Very low |  |
| *Global state - Amineptine* | | | | | | | | | | | | |
| 2 | Randomised trials | Serious^c^ | Not serious | Not serious | Serious^d^ | None | 36 | 36 | - | MD **0.49 lower** (0.8 lower to 0.17 lower) | ⨁⨁◯◯ Low |  |
| **Withdrawal symptoms** | | | | | | | | | | | | |
| 3 | Randomised trials | Not serious | Not serious | Not serious | Very serious^b^ | None | 43 | 50 | - | SMD **0.17 higher** (0.43 lower to 0.77 higher) | ⨁⨁◯◯ Low |  |
| **Craving** | | | | | | | | | | | | |
| 2 | Randomised trials | Serious^c^ | Not serious | Not serious | Very serious^b^ | None | 24 | 24 | - | SMD **0.34 higher** (0.77 lower to 1.45 higher) | ⨁◯◯◯ Very low |  |
| **Safety** | | | | | | | | | | | | |
| 1 | Randomised trials | Very serious^g^ | Not serious | Not serious | Very serious^b^ | None | 7/17 (41.2%) | 3/18 (16.7%) | **RR 2.47** (0.76 to 8.03) | **245 more per 1000** (from 40 fewer to 1000 more) | ⨁◯◯◯ Very low |  |

CI, confidence interval; MD, mean difference; RR, risk ratio; SMD, standardised mean difference.

a. Three of the included studies were judged to be of high risk of bias across multiple domains.

b. The 95% confidence interval crosses the threshold for clinical decision making, and the optimal information size was not met for this outcome

c. One of the included studies was judged to be of high risk of bias across one domain.

d. The optimal information size was not met for this outcome.

e. One of the included studies was judged to be of high risk of bias across multiple domains

f. The results for this outcome were significantly and substantially heterogeneous.

g. The included study was judged to be of high risk of bias across multiple domains.
